# The Adoption, Implementation, and Impact of Smoke-Free Policies among Gaelic Athletic Association Clubs in Ireland: A Qualitative Study

**DOI:** 10.3390/ijerph17051785

**Published:** 2020-03-10

**Authors:** Christopher M. Seitz, Jennifer Lawless, Stacey Cahill, Aoife O’ Brien, Collette Coady, Colin Regan

**Affiliations:** 1 Appalachian State University, Department of Health & Exercise Science, Boone, NC 28608, USA; lawlessja@appstate.edu; 2 Gaelic Athletic Association, Community & Health Department, Croke Park Stadium, D03 P6K7, Dublin 3, Ireland; stacey.cahill@gaa.ie (S.C.); aoife.obrien@gaa.ie (A.O.B.); collette.coady@gaa.ie (C.C.); colin.regan@gaa.ie (C.R.)

**Keywords:** smoking, tobacco, policy, sports, Ireland

## Abstract

The Gaelic Athletic Association (GAA) is a major stakeholder in promoting smoke-free policies in Ireland. Several GAA clubs have adopted smoke-free policies, and there is a growing interest among other GAA clubs to also become smoke-free. As such, the purpose of this study is to explore the process of how GAA clubs adopt, implement, and enforce smoke-free policies in order to discover best practices that other clubs could replicate. Representatives from 15 smoke-free clubs were interviewed regarding how their club became smoke-free. Interview data were analyzed, in which four major themes emerged: (1) process (planning a smoke-free policy, communicating the policy to the community, providing smoking cessation resources), (2) barriers (opinions and behaviors of club members who smoke, bars connected to club houses, policy exceptions, visitors and umpires who were unaware of the policy), (3) enforcement (community-based style of enforcement, non-confrontational approach, non-enforcement), and (4) impact (observation of policy compliance and decrease in cigarette litter). The study’s findings indicate a general ease of becoming smoke-free with minimal barriers. As such, the GAA should urge each club to become smoke-free and to use the effective methods used by current smoke-free clubs for communicating and enforcing smoke-free policies.

## 1. Introduction

Smoking is a major public health problem in Ireland. It is the leading cause of preventable death, killing over 5000 Irish people each year, which is nearly one out of every five deaths [1,2]. Each year, smoking costs Ireland 69 million Euro (EUR) in litter, EUR 6 million in fires, EUR 506 million in healthcare, EUR 1071 million in lost productivity, EUR 1355 million in morbidity, and EUR 7657 million in mortality [2]. According to the 2018 Healthy Ireland survey, 20% of the population currently smoke [3].

An evidence-based approach to reducing and preventing smoking is to implement smoke-free policies. The World Health Organization recommends the implementation of policies that ban smoking in indoor and outdoor areas [4]. Fortunately, in 2004, Ireland became the first country to ban smoking in the workplace, indoor public places, restaurants, public houses (bars), education buildings, healthcare facilities, and any form of public transportation. In terms of outdoor smoking policies, Ireland’s Department of Health is currently working to promote smoke-free university grounds, hospital and government grounds, and sporting facilities [1].

A key stakeholder in promoting smoke-free policies in Ireland is the Gaelic Athletic Association (GAA). The GAA was founded in 1884 in order to encourage Irish people to play sports that were traditional and indigenous to Ireland. Today, there are over 1600 GAA “clubs” located across each of Ireland’s 32 counties, and another 400 international clubs set up by Irish diaspora. Clubs are located in Irish communities and consist of administrators, community volunteers, athletes, and sporting grounds that promote the sports of hurling, Gaelic football, handball, rounders, ladies football, and camogie among all age groups. Clubs also encourage Irish culture, including the use of Irish music, song, dance, and the Irish language. In all, GAA clubs are an influential force within Irish society [5,6].

In 2013, the GAA’s Community and Health Department (CHD) began the Healthy Club Project (HCP). The GAA’s HCP has partnered with Healthy Ireland, the Health Service Executive (HSE), the National Office for Suicide Prevention, with Irish Life as corporate social responsibility partners, with an aim to “make every GAA club in Ireland a hub for health, capable of providing their members and communities with programs that support their physical, emotional, and social wellbeing” [7]. One of the HCP’s objectives is to advocate for clubs to adopt policies that prohibit smoking outdoors on club premises in order to reduce second-hand smoke exposure and de-normalize smoking in club settings [8]. In 2018, there were a total of 28 smoke-free GAA clubs [9].

The HCP’s goal of GAA clubs becoming smoke-free is in alignment with the European Healthy Stadia Network’s recommendation for smoke-free stadiums. The European Healthy Stadia Network (also known as “Healthy Stadia”) was formed during the 2000s by several sports stadiums in the United Kingdom that were implementing public health programs and policies to improve the health of players, fans, and surrounding community members. This movement was later funded by the EU’s public health programme and formally established Healthy Stadia with the purpose of advocating and providing resources, for sports stadia to become environments that promote health [10]. One of Healthy Stadia’s major efforts is advocating for stadiums to become totally smoke-free [11].

Unfortunately, there is limited research regarding the process of how sporting facilities become smoke-free. Several studies have assessed the public’s approval of smoke-free stadiums in various countries [12,13,14,15], and other studies have assessed non-smoking signage and spectator compliance within smoke-free stadiums [16,17,18,19,20]. However, only two studies have investigated the process of how stadiums become smoke-free. In the first study, researchers interviewed staff from smoke-free stadiums and from Australia’s Victorian Health Promotion Foundation, finding that an increase in smoke-free stadiums in Australia was associated with increased efforts from health professionals to advocate to stadium administrators to adopt smoke-free policies through educational seminars and one-on-one meetings [21]. The second study described how Healthy Stadia worked with the Union of European Football Association (UEFA) to make the entire 2016 European Championships in France a smoke-free sporting event. This case study found that it was important to assess the stadium for “hot spot” areas where smoking could take place, thoroughly communicate the smoking policy to spectators, and train staff in how to softly enforce the policy by giving informational cards to policy violators [11].

Given the strong influence of GAA clubs in Irish communities, the growing interest of GAA clubs to become smoke-free, and the general lack of research on this topic, there is a need to study how smoke-free policies have been adopted by GAA clubs. By doing so, best practices can be discovered for other clubs to effectively transition to becoming smoke-free. As such, the purpose of this study is to explore the process of how GAA clubs adopt, implement, and enforce smoke-free policies, while also discovering any barriers that clubs experienced in that process.

## 2. Materials and Methods 

Club representatives who were familiar with how their club became smoke-free were recruited via e-mail or telephone from contact information that was provided by the GAA HCP. Individuals from 15 smoke-free clubs agreed to participate in the study. The participants included nine health and wellbeing officers, three healthy club committee members, two chairman of healthy clubs, and a club treasurer. Those that participated in the research study were from clubs that reflected a broad representation of all the GAA’s clubs in Ireland in terms of location and size. Specifically, the clubs were from urban, rural, and suburban areas, with a membership bridging from the hundreds to the thousands. Before recruitment and data collection took place, the Institutional Review Board (IRB) at Appalachian State University, in Boone, North Carolina, reviewed and approved the ethics of the research project’s methods.

Interviews were conducted one-on-one, in person and by telephone. The interviews were recorded and then transcribed verbatim. All identifying information was not included in the transcriptions. The study’s interview questions were formed through discussion with the GAA HCP leadership and included the following items: (1) How did your GAA club became smoke-free?, (2) Did the club experience any barriers when trying to become smoke-free?, (3) How do you communicate your smoke-free policy and any smoking cessation resources?, (4) How have the members in your club felt about the club becoming smoke-free?, (5) Do you think the policy has been effective in reducing smoking in the clubs?, (6) Do you think that people are complying with the policy?, and (7) How is the policy enforced? 

After transcribing the data, two researchers immersed themselves in the data by reading the interview transcripts several times during and after data collection [22]. During this immersion process, the researchers conducted “crystallization,”, which included pausing from being immersed in the data in order for the researchers to discuss and define the themes that were emerging from the data [22]. During this process, the researchers also determined when saturation took place (the moment when different, distinct themes no longer emerge from data) [23], which happened at the 12th interview. Then, illustrative statements from the data were clustered into the themes, providing both a structural meaning (how clubs became smoke-free) and also a textural description (what happened when the clubs became smoke-free) [24]. This process was not conducted using qualitative software; instead, the analysis was performed on a Microsoft Word document by coding highlighted text that reflected each theme.

## 3. Results

There were four distinct themes that emerged from the data, including Process, Barriers, Enforcement, and Impact.

*Theme #1: Process*

Overall, participants described a common process of adopting a smoke-free policy. This process tended to include joining the HCP, forming a committee, and then discussing a smoke-free policy among committee members and club leadership. Some of the clubs did not seek outside resources in this process:

*“We first joined the Healthy Club [Project] group, and then as part of that, we decided to become smoke-free. So to do that, we got buy in from members, club executive, and basically plowed on from there.” - Participant #1*

*“We would have monthly meetings as a committee like and going completely smoke free had come up a few times and then we just decided that we were going to do it.” - Participant #14*

*“…we discussed ideas just at the health and wellbeing committee, just discussed it (going smoke-free) causally. Just at our own committee stage and then in November, we brought it to the Senior Executive of the club and we discussed it there and weighed out the pros and cons and discussed problems that could happen. You know the impact that it would have on the older members of the club who have been coming along to the games for years and then suddenly have this (smoke-free) policy thrown upon them. So, we did debate it out and revisited even just causally among members after that, and then with a plan in a May to go through with it.” - Participant #7*

Several clubs used available resources when developing a plan to adopt a smoke-free policy. These resources included encouragement from the GAA’s HCP leadership, other smoke-free clubs, and local general practitioners:

*“We formed a committee and the majority of the votes were in favour for the smoke-free club, so then we just followed the guidelines given to us by the Healthy Club (Project).” - Participant #11*

*“We were the first (club) in our own county to do it, so I suppose we just put a plan together ourselves. We had our own little team we put together and I would have received some kind of information from St. John’s down in Wexford at the time. I was chatting to the guys there on how they implemented it (smoke-free policy) on their end, you know just did up a rough plan of how we were going to implement it then and basically get approval before we could go forward from it…We would have had the resource there from the wellbeing team in Croke Park. There was a few clubs at the time that were doing it as well, so we were able to bounce ideas off each other.” - Participant #9*

*“So, we first applied to be a Healthy Club and, once we got approved, we decided that one of our goals would be to go smoke-free. One of the things we did was reach out to the local GP (general practitioner) and pharmacies. So, they would have been on board along with the GAA committee and we created our own committee for the healthy club and we voted on the goal of smoke-free.” - Participant #14*

After adopting a smoke-free club policy, several clubs then communicated the new policy to club members and the local community. There were a variety of ways that clubs communicated the policy, including the use of social media (e.g., Facebook, Twitter), using the intercom during matches, sending e-mail and text messages to club members, handing out cards/flyers, posting smoke-free signs provided by the GAA, relying on word-of-mouth, and communicating through local newspapers:

*“We had one big match and we just said on the intercom there that we were a smoke-free club and to go (smoke) outside the gates.” - Participant #2*

*“We gave out little cards at the gate to remind people that the park is smoke-free. We also partner up with the visiting team and get them to put up a notice on their Facebook wall as a reminder that the club is smoke-free.” - Participant #6*

*“First of all, we focused on total membership by texting the members and using the Facebook wall. We were also on the local papers.” - Participant #1*

*“We had a combination of using social media and then sending out messages to our members. As for now, it’s more of we have the (smoke-free) signage up...” - Participant #11*

In addition, several clubs hosted a “launch” event to draw attention to the policy from club members, communities, and the media. Some of the launch events included having a ribbon-cutting ceremony, inviting speakers to give presentations, and hosting an anti-smoking coloring competition for young club members:

*“Like we had a launch date and made a big thing of it. So, we got the minister to come down and Aoife from the Healthy Club [Project] in Croke Park came and we got other people from the HSE to come and the local doctor and we had a launch. They each spoke and gave a little presentation. We involved the school as well; we had an art competition.” - Participant #2*

*“When it was implemented the HSE then got involved along with a few other groups and they set up a few stalls on the day of the launch and had displays of how smoking affected people’s health. We also had a local doctor come along and provide a medical opinion on how smoking negatively affects the body.” - Participant #13*

For several clubs, part of the process of adopting and implementing a smoke-free policy was also to communicate smoking cessation resources to those who smoke, including online resources and quit-lines. However, a few participants stated that there was a low level of interest by smokers in terms of accessing smoking cessation resources:

*“The Healthy Club [Project] has given us lots of information, posters, and cards on quitting. The local GP and pharmacies are big into it and have lots of support. They always have cards with ways to quit and things. But, yeah, we do have numbers up in the club house and national quit line up on the board as well. So the numbers are there if they want them along with cards if they want to quit.” - Participant #3*

*“We have it [smoking cessation information] on the wee cards as well. It says we are smoke-free, thank you for not smoking. For more information on quitting, go to wanttostop.info. So we have that on the wee card. We have on the social media resource along with other things. There’s a link under health and wellbeing; there’s a whole list of telephone numbers for quitting tobacco and alcohol along with other things.” - Participant #6*

*“We did consider running a smoking cessation course through the HSE but we didn’t get much of an uptake from it.” - Participant #14*

*Theme #2: Barriers*

Regarding barriers to becoming a smoke-free club, several participants expressed that they did not experience any barriers to overcome in order to adopt a smoke-free policy:

*“…we didn’t really come to any barriers when implementing the club. Everyone thought it was a good idea to go smoke-free since we were joining the healthy club program.”*

*- Participant #1*

*“When we put it (the smoke-free policy) to the committee, there were no problems, they thought it was a great idea to go smoke-free.” - Participant #2*

*“We thought getting through the committee meeting would be harder because there are some smokers in the club. But, no, there wasn’t too much fuss about us going smoke-free.”*

*- Participant #3*

Of the clubs that did experience barriers, a common experience was the opinions and behaviors of club members who smoke. There were a few specific populations that were mentioned in the interviews, including smokers who were older in age, caretakers, and others:

*“…we have a number of old folk events which would include card night and an afternoon kind of event for them and they would be smokers and they are not going to stop smoking like, so, we knew that was always going to be a problem.” - Participant #15*

*“The caretakers were a barrier; we knew they would be the most difficult part. But, we didn’t really allow them to have a say in anything so it wasn’t too much of a barrier. They go outside now to smoke and its fine.” - Participant #5*

*“We had one or two people who weren’t in agreement within our own club who said they find it very frustrating, like, they would be great supporters of the GAA, and the thought of them going to a match and not have their one or two cigarettes there.” - Participant #12*

Another barrier was the presence of bars that are connected to clubhouses. One participant noted the difficulty of having a smoke-free policy when people are drinking at the bar and go outside of the bar to smoke:

*“The only time that you see smoking in our club is when there is an event on when the bar is open and you’ll see people go out for a puff then, but generally speaking, you wouldn’t see it in our club.” - Participant #8*

One participant also stated that barriers to a smoke-free policy were the possibility of making exceptions to the policy by allowing smoking in certain zones in the club or allowing the use of e-cigarettes. At the time of the study, e-cigarette use was not banned in public places, unless an organization voluntarily banned e-cigarettes on its premises:

*“When we would discuss it, people would be saying, ‘Oh, you know, are we going to have a smoking zone? Like, it’s not fair now for people to come up here and have nowhere for people to smoke.’ Just sort of negative little digs. So, to overcome them, the first couple of times I was going like, ‘Yeah, you’re right, maybe you’re right, maybe we should have a smoking zone.’ But then, the more I thought about it, we have agreed to be a healthy club and be a part of the initiate and it’s for the good of everybody and 99% people support it. The vast majority of people want it so you just have to go with the majority and will what you think it is right.” - Participant #7*

*“The only thing that has come up is the vaping. Can you vape? But, I had to think on my feet a little and just said no to the vaping as well, because it’s about the image and the message you’re sending out as well. No smoking of anything in the club.” - Participant #7*

Club representatives expressed that a common barrier to their smoke-free policy was visitors and umpires who are unaware of the policy; however, when politely confronted, most visitors become compliant to the policy and stop smoking:

*“We have had a few visiting people that have persisted smoking, but more so because they didn’t notice the signs, and when brought to their attention that we were a smoke-free club, they stopped.” - Participant #1*

*“...I find that most people (who violate the policy) are people from other teams and although there are signs up, they don’t actually notice them, and then straight away, they are apologetic and say they didn’t notice.” - Participant #2*

*“There was a visiting team and the umpire was smoking at the goal post and he politely asked to put it out and he gave back some verbal abuse. It was one of our club members who asked, and she just said, ‘You know this is a smoke-free club, do you mind putting it out?’ And he not so politely said, ‘Go away.’ But, he did eventually did put it out.” - Participant #6*

*“…like there was an umpire smoking there just the other day and I had to approach him and tell them, and in fairnesss, he just didn’t know so there were no problems.” - Participant #15*

*Theme #3: Enforcement*

Several clubs enforced their smoke-free policy through a community-based style of enforcement. Specifically, the entire community of players, coaches, administrators, and parents were expected to kindly inform those who smoke that the club is a smoke-free environment:

*“Most parents and even the kids now are aware of it now so if someone does smoke on site, someone is quick enough to come to one of the committee members or approach themselves and tell them (the policy violator) that this is a smoke-free zone, and so it’s been very positive. I have even seen a child go up to someone smoking and telling them that they aren’t allowed to do that in the club. So, I think it has been very effective.” - Participant #1*

*“It’s (enforcing the policy) just really on ourselves. If someone sees someone smoking, they will say something to them. I know there myself I saw somewhere there recently and said something, but like there’s never a huge problem with it.” - Participant #5*

*“Generally any of the members, if they see anyone smoking they just say we’re smoke-free, you know. Just everyone’s responsibility.” - Participant #14*

Others stated that only the Healthy Club committee had the responsibility for enforcing the policy:

*“Well, there is a committee of about 20 and we made it primarily their business to enforce it.” - Participant #6*

*“It’s the Healthy Club committee really so we take it (enforcing the policy) in turns, depending how big the match on is. For the smaller events, you’d only need two or three.”*

*- Participant #2*

The clubs that enforced their smoke-free policy also clarified that when people enforced the policy, they were intentionally non-confrontational and very polite to those who violate the policy:

*“When we do (enforce the policy), it’s just kind of like a gentle reminder.” - Participant #10*

*“We are no smoking and we are not going to ram it down your throat. We are going to carefully encourage it and make people aware of it.” - Participant #12*

*“We all said that it would be the responsibility of everyone is the club from the players, parents, and committee members to just politely just say to people, ‘Sorry, don’t know if you are aware, but this is a non-smoking club.’” - Participant #9*

On the other hand, there were clubs that did not actively enforce their smoke-free policy, which may have been due to people feeling uncomfortable approaching smokers to inform them of the policy:

*“Well, you see, initially, we would have gone up to people but we haven’t really in the past year or so. We don’t have smoke wardens; even if we see someone lighting up, we won’t challenge them. It’s kind of a voluntary thing.” - Participant #4*

*“Am I going to go up to somebody, and ask them to put out their cigarette? I’m probably not going to do that, but I would hope that they wouldn’t in respect for the policy and what we are trying to do. And you know you will always have somebody who is going to break the rules, but that’s life.” - Participant #7*

*“And you know not everyone is comfortable saying, like, ‘Would you mind not smoking around me?’ Because it can start an argument.” - Participant #12*

*Theme #4: Impact*

Based on personal observations, those who were interviewed noticed that most people were complying with the smoke-free policy:

*“Most of the members have been very supportive. Members haven’t smoked on-site, as far as I am aware.” - Participant #1*

*“Most of the time, I would say 98% of the people are in favor of it (the smoke-free policy). We have had very little complaints or objections. You know everyone, there are a lot of smokers, but they do seem to go away or sit in their own cars to smoke because they know the club is smoke-free.” - Participant #3*

*“Now, there’s no point in saying that it would be completely smoke-free zone, that is not the case. You will some people lighting up, but it (the smoke-free policy) has definitely reduced it.” - Participant #4*

As an additional indication of reduced smoking, the participants also noticed a reduction in the amount of littered cigarette butts on the club’s grounds:

“…that’s becoming more evident than at the start, there would be certain areas where there would be cigarette butts, but now it is less and less. I don’t know when the last time I saw any (cigarette butts), and I go in there (the club grounds) every evening.” - Participant #1

“So, as for the grounds, the litter has decreased, but there’s still some outside of the gates.”

- Participant #2

“Oh yeah, there has definitely been a reduction in the (cigarette) litter.” - Participant #10

## 4. Discussion

Several practical findings resulted from exploring how GAA clubs adopt, implement, and enforce smoke-free policies. First, given the ease that most clubs experienced in transitioning to a smoke-free club, and given the effectiveness of such a policy, the GAA HCP should urge each club to become smoke-free. Although only a small sample of smoke-free clubs were included in the study, it can be assumed that other clubs that become smoke-free would experience similar positive effects in regards to adopting a smoke-free policy, as well as observing a decrease in smoking behavior and second-hand smoke exposure. For the most part, the clubs involved in this study experienced a straightforward transition in regards to becoming smoke-free. Club leaders observed strong support from within the club, support from resources outside the club, and minimum barriers in the process of becoming smoke-free. As such, this study suggests that any apprehension about pushback from the community members within GAA clubs may be unfounded. Moreover, clubs considering smoke-free policies may plan ahead of time to develop a plan to deal with common potential barriers.

Second, it appears that current methods used by clubs for communicating and enforcing smoke-free policies are effective. Specifically, clubs considering the adoption of a smoke-free policy should consider hosting a smoke-free launch event that includes the community, especially young club members, media outlets, and well-known local people (i.e., general practitioner physicians). Clubs should also plan to use established methods to inform the community and visitors about the smoke-free policy (e.g., smoke-free signs, social media, e-mail, flyers, coaches/teams, intercom announcements). Thorough communication of a new smoking policy was also echoed by Healthy Stadia in its case study of the smoke-free 2016 UEFA European Championships that were held in France [11].

In terms of policy enforcement, potential smoke-free clubs should plan to use a friendly, non-confrontational method of enforcement carried out by club leaders and/or the entire club community. Although previous international research indicates that those who attend sporting events in smoke-free stadiums tend to comply with the policy [17,19,20], enforcement for those who do not comply is important to protect others from second-hand smoke exposure. The soft, friendly, informational enforcement approach found in this study, and that of Healthy Stadia [11], is also a method of enforcement used at smoke-free universities in the United States [25,26,27]. 

Finally, potential, and already established, smoke-free clubs should consider making smoking cessation resources available to club community members. Although the findings suggest that smoking cessation resources were not popular among those trying to quit smoking, one of the World Health Organization’s recommended evidence-based strategies to increase quit attempts within a community is warning people about the dangers of smoking and also making cessation resources available [28]. 

The limitations of the study’s methodology should be considered when interpreting the findings. As with all qualitative data, the themes that emerged from the interviews cannot be generalized to all GAA clubs; however, qualitative findings may be “transferable” [29], meaning that other GAA clubs might experience similar effects shown in the study’s results.

## 5. Conclusions

The findings from this study strongly suggest that there is a general ease of GAA clubs becoming smoke-free, and that smoke-free policies are effective and fairly easy to enforce. As such, given the harmfulness of tobacco smoke and its negative impact on Ireland, the GAA should urge each club to become smoke-free and to use the methods used by current smoke-free clubs for communicating and enforcing smoke-free policies. Ultimately, this would help to support the vision of the Irish government for a healthy, smoke-free Ireland [1]. From a broader perspective, this study also adds to other research [11,21] regarding best practices in the process of clubs/stadiums outside of the GAA becoming smoke-free.
